# Clinical Outcome and Comparison of Regenerative and Apexification Intervention in Young Immature Necrotic Teeth—A Systematic Review and Meta-Analysis

**DOI:** 10.3390/jcm11133909

**Published:** 2022-07-05

**Authors:** Pratima Panda, Lora Mishra, Shashirekha Govind, Saurav Panda, Barbara Lapinska

**Affiliations:** 1 Department of Conservative Dentistry and Endodontics, Institute of Dental Sciences, Siksha ‘O’ Anusandhan University, Bhubaneswar 751003, Odisha, India; pandapratima085@gmail.com (P.P.); shashirekhag@soa.ac.in (S.G.); 2 Department of Periodontics and Oral Implantology, Institute of Dental Sciences, Siksha ‘O’ Anusandhan University, Bhubaneswar 751003, Odisha, India; sauravpanda@soa.ac.in; 3 Department of General Dentistry, Medical University of Lodz, 92-213 Lodz, Poland

**Keywords:** apexification, endodontic therapy, immature permanent tooth, pulp, regeneration

## Abstract

This systematic review aimed to evaluate interventions individually and compare the clinical outcome of young, immature teeth treated with regenerative endodontic therapy (RET) and apexification procedure. The protocol was registered with PROSPERO (International Prospective Register of Systematic Reviews), bearing the registration number CRD42021230284. A bibliographic search in the biomedical databases was conducted in four databases—PubMed, CENTRAL, EMBASE and ProQuest—using searching keywords and was limited to studies published between January 2000 and April 2022 in English. The search was supplemented by manual searching, citation screening and scanning of all reference lists of selected paper. The study selection criteria were randomized clinical trial, prospective clinical studies and observational studies. The search found 32 eligible articles, which were included in the study. The quality assessment of the studies was performed using the Cochrane risk of bias tool for randomized control trials and non-randomized clinical studies. The meta-analysis was performed using Review Manager software (REVMAN, version 5). The results indicated that a clinicians’ MTA apexification procedure was more successful compared to calcium hydroxide. In RET, apical closure and overall success rate is statistically same for both apical platelet concentrates (APCs) and blood clots (BC). Both interventions have similar survival rates; however, RET should be preferred in cases where the root development is severely deficient, there is insufficient dentine and the tooth’s prognosis is hopeless even with an apexification procedure.

## 1. Introduction

In permanent dentition, traumatic dental injuries (TDI) are a worldwide health issue and the most frequent cause of pulpal necrosis [1]. In 85% of TDIs, patients have injuries to the oral region [2]. Globally, around one billion people are affected by trauma [3], and one-third of these patients have injuries to their immature teeth that might cause pulp necrosis [4].

Pulp necrosis due to trauma or caries in children and adolescents may hinder permanent tooth root growth, resulting in thin dentinal walls, wide-open apices, and an insufficient crown:root ratio [5]. According to Cvek, the classification of root development in an immature necrotic permanent tooth can be at stage 1, where less than half of the root formation with open apex is present; stage 2 is where half of root formation with open apex is present; and stage 3 is when 2/3 of root development with open apex is present [6].

In conventional root canal fillings, immature permanent teeth with necrotic pulps are difficult candidates and have an increased susceptibility to root fractures after treatment [7]. Hence, early intervention for non-vital immature teeth is critical. However, it is incredibly challenging, time-consuming and technically complex [8]. Apexification and regeneration are interventions routinely practiced in such cases [9]. RET is recommended in short roots with thin canal walls, a wide-open apex and for teeth lacking the potential for root formation, whereas apexification is done in the tooth which has nearly completed root formation with an open apex [5].

Apexification is a method to encourage the development of an apical barrier to close the open apex of an immature necrotic permanent tooth in which filling materials can be placed within the root canal space [10].

In contrast, RET or regenerative endodontic procedures (REPS) are biologically based procedures designed to replace damaged structures, such as the root and dentin, along with cells of the pulp–dentin complex [11]. The main aim of REPS is to establish a suitable environment (biomimetic microenvironment) in the root canal to facilitate mesenchymal stem cells such as osteo/odontoprogenitor stem cells, pulp tissue regeneration and continued root development.

The basic principles underlying both interventions involve removing necrotic pulp, debridement of the canal and control of infection with or without antiseptic medicament. Total treatment time may vary in multi-visit apexification, depending on the medicament used, the initial presence of periapical pathology [6], the frequency of medicament replacement [12], and the age of the patient [13].

This present review compares and assesses both interventions individually to manage immature necrotic young permanent teeth. This review aims to critically evaluate the outcome of regeneration and apexification procedure, which will impact clinical discussion making.

## 2. Materials and Methods

The review protocol was registered at PROSPERO (International Prospective Register of Systematic Reviews), bearing registration number CRD42021230284. This review followed the Preferred Reporting Items for Systematic Reviews and Meta-analyses (PRISMA) statement guidelines [14].

### 2.1. Search Strategy

The following PICO components were established: Population (P)—systematically healthy patients with necrotic young, immature permanent tooth; Intervention (I)—regeneration procedure; Comparison (C)—apexification procedure; Outcomes (O)—clinical and radiographical successful outcome. The research question was: “Which intervention between and within the two that is regenerative and apexification, has a more successful outcome in the young permanent non-vital tooth?**”**

The electronic search strategy is described in Table 1. A comprehensive electronic search for relevant articles was performed in the PubMed, CENTRAL, EMBASE and ProQuest databases using the search keywords and combining the keywords using “AND” and “OR”. For all these databases, Boolean operators (OR, AND) were used to combine and narrow down searches that included appropriate MeSH terms, keywords, and other terms following the syntax rules of each database. All references selected in the search were saved in Mendeley Desktop software to remove the duplicates.

A manual search was performed in the following dental journals: *International Endodontic Journal*; *British Dental Journal*; *Journal of Endodontics*; *Oral Surgery, Oral Medicine, Oral Pathology, and Oral Radiology*; and *Endodontics*. The search was supplemented by manual searching, citation screening and scanning all reference lists of the selected paper. Additional studies that were likely suitable for inclusion were screened from the bibliographies of potentially eligible clinical trials, case reports, case studies, and systematic reviews.

### 2.2. Study Selection

The literature search was limited to articles available in English and to those published between January 2000 and April 2022. Each article was assessed carefully and in detail. Two independent reviewers (P.P. and L.M.) read abstracts and titles, and studies not about the research question were excluded. The remaining relevant studies’ full texts were read and analyzed independently. In this selection, a third reviewer (S.G.) was called to achieve a consensus if there was a disagreement of opinions.

The selection of studies was performed with no restrictions on place or year of publication. However, a language restriction was applied, and only those articles written in English were included. Titles and abstracts were analyzed to determine whether they fulfilled the inclusion criteria. The inclusion and exclusion criteria are depicted in Table 2.

The relevant data of the included trials were extracted in detail using an Excel spreadsheet (Microsoft, Redmond, WA, USA) independently by two review authors (P.P., L.M.) and recorded in spreadsheets. In case of missing or unclear information, the authors of the included reports were contacted by email to provide clarification regarding data given or any missing information. The data of all included studies were entered in the characteristics of included studies tables in Review Manager (RevMan, Version 5).

### 2.3. Study Quality Assessment

Two review authors (P.P., L.M.) independently assessed the risk of bias in the included studies. In case of disagreement, a third review author (S.G.) was consulted. For the randomized control trials, the assessment was conducted following the instructions and the approach described in the Cochrane Handbook for Systematic Reviews of Interventions [15].

For each study, the following domains were considered: selection bias (random sequence generation and allocation concealment), performance bias (blinding of participants and personnel), detection bias (blinding of outcome assessment), attrition bias (incomplete outcome data addressed), and reporting bias (selective reporting).

For the non-randomized controlled trial, the risk of bias in included studies was assessed using the ROBINS-I risk of bias tool. The bias tool considered: bias due to confounding, selection of participants, classification of interventions, deviations from intended interventions, missing data, measurement of outcomes and selection of reported results.

The overall risk for individual studies was assessed as low, moderate, serious or critical based on the following criteria: low or moderate risk of bias if all domains were at low risk of bias; serious risk of bias if at least one domain was at serious risk of bias but not at critical risk of bias in any domain; critical risk of bias if one domain was at critical risk of bias.

## 3. Results

### 3.1. Selection of Studies

**Figure 1:**
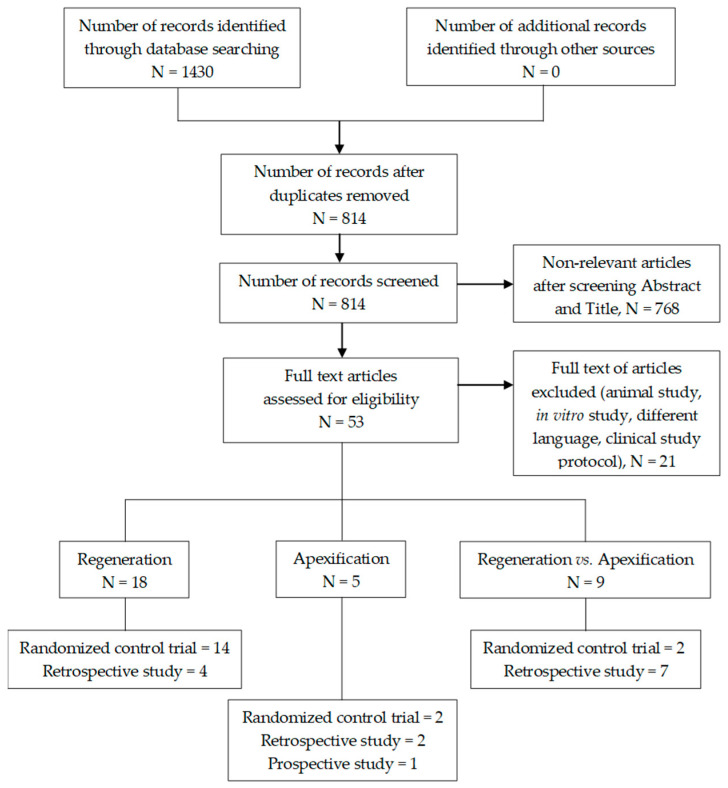
PRISMA 2020 flow diagram for systematic review that includes searches of databases.

The search in the selected databases allowed for the identification of 1430 articles (Figure 1). After eliminating duplicates, the included articles were selected from a pool of 814 articles obtained from digital sources and a manual search. The full text was read for 53 articles, and 21 studies were omitted for the reasons specified in Appendix A (Table A1). A total of 18 randomized clinical trials and 14 non-randomized clinical trials were included in this systematic review to assess the successful outcomes in managing immature young necrotic permanent teeth.

### 3.2. Characteristics of Studies

#### 3.2.1. Design

Thirty-two articles were included in this study (Table 3). Eighteen articles [16,17,18,19,20,21,22,23,24,25,26,27,28,29,30,31,32,33] evaluated the clinical outcome of regenerative endodontic procedures (REP) and five articles [34,35,36,37,38] on the apexification procedure. Only nine articles [39,40,41,42,43,44,45,46,47] evaluated and compared the clinical outcome between regeneration and apexification procedure.

#### 3.2.2. Participants

In total, of the 18 articles [16,17,18,19,20,21,22,23,24,25,26,27,28,29,30,31,32,33] evaluating regeneration outcomes, 14 articles [16,17,18,19,20,21,22,23,24,25,26,27,28,33] were randomized clinical trials, and 4 articles [29,30,31,32] were non-randomized clinical articles. In randomized clinical trials, a total of 393 participants and 412 teeth were included. In non-randomized clinical trials, 144 participants and 156 teeth were included.

Five articles [34,35,36,37,38] evaluated apexification outcomes, of which two articles [34,35] were randomized clinical trials and three [36,37,38] were non-randomized clinical trials. In RCT, a total of 68 participants and 68 teeth were included. In NRCT, a total of 198 participants and 200 teeth were included. One NRCT article was a multicentric study.

Nine articles [39,40,41,42,43,44,45,46,47] compared the outcome between regeneration and apexification procedure, of which two articles [39,40] were RCTs, and seven articles [41,42,43,44,45,46,47] were NRCTs. In the RCTs, a total of 133 participants and 133 teeth were included. In the NRCTs, a total of 439 participants and 446 teeth were included.

#### 3.2.3. Intervention

Of the total eighteen RCTs, four studies [16,17,18,19,20,21,22,23,24,25,26,27,28,33] evaluated the revascularization procedure outcomes, two studies [34,35] were on the apexification and two studies [39,40] compared revascularization versus apexification.

Among fourteen NRCTs, four studies [29,30,31,32] investigated clinical outcome of the revascularization procedure, three studies [36,38] were on apexification and seven studies [41,42,43,44,45,46,47] compared regeneration versus apexification

### 3.3. Analysis of Quality of the Studies

**Figure 2:**
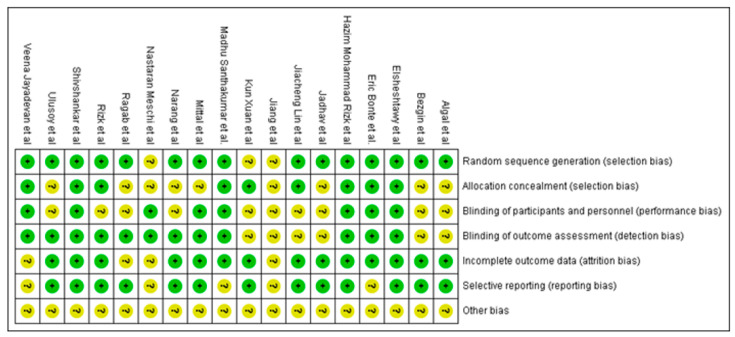
Quality assessment of included RCT studies summary: review authors’ judgements about each risk of bias item for each included study.

**Figure 3:**
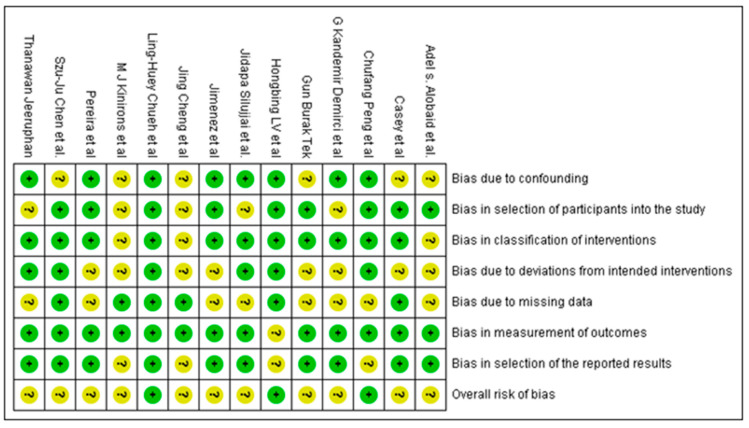
Quality assessment of included NRCT studies summary: review authors’ judgements about each risk of bias item for each included study.

The risk of bias in included studies is summarized in Figure 2 for RCTs and Figure 3 for NRCTs.

In regeneration RCTs, two studies [18,24] were assessed to be at low risk, whereas twelve studies [16,17,19,20,21,22,23,25,26,27,28,33] were at moderate risk of bias. In apexification RCTs, two studies [34,35] were assessed to be at moderate risk of bias. In regeneration versus apexification RCTs, two studies [39,40] were assessed to be at moderate risk of bias.

In most of the randomized clinical trials, there was unclear or no information about allocation concealment, blinding of participants and blinding of outcome evaluation in a few studies. The factors mentioned above resulted in a moderate overall risk assessment in the studies cited above. In most non-randomized control trials, there was unclear or no information on sample selection, exact treatment protocol and deviations from planned interventions in a few studies. The variables mentioned above resulted in a moderate to serious overall risk assessment.

In regeneration NRCTs, one study [32] was assessed to be at low risk of bias whereas three studies [29,30,31] were at moderate risk. In apexification NRCTs, one study [37] was assessed to be at moderate risk of bias and two studies [36,38] were assessed to be at serious risk. In regeneration versus apexification NRCTs, one study [47] was assessed to be a low risk of bias, five studies [42,43,44,45,46] were assessed to be a moderate risk, and one study [41] was at serious risk.

### 3.4. Synthesis of Results

The meta-analysis (Review Manager, RevMan version 5.3, Copenhagen: Nordic Cochrane Centre, The Cochrane collaboration) was performed with quantitative outcome data extracted from the six included randomised controlled trials in REP, which compared the effectiveness of APCs in comparison to BC for treatment of young, immature, necrotic, permanent teeth. However, it was not possible in case of the NRCTs, as the data from the included studies showed heterogeneity (Table 4).

Forest plots were plotted individually in a random effect model for dentinal wall thickness (DWT), increase in root length (RL), apical closure (AC), vitality response (VR) and success rate (SR). Meta-analysis was also performed to compare REP and Apexification procedure. The meta-analysis was made from six included trials. Forest plots were plotted for survival rate (SR), success rate (SR), increase in root length (RL) and decrease in foramen width (FW).

#### 3.4.1. DWT in REP with APC Compared to BC in Young Immature Permanent Teeth

**Figure 4:**
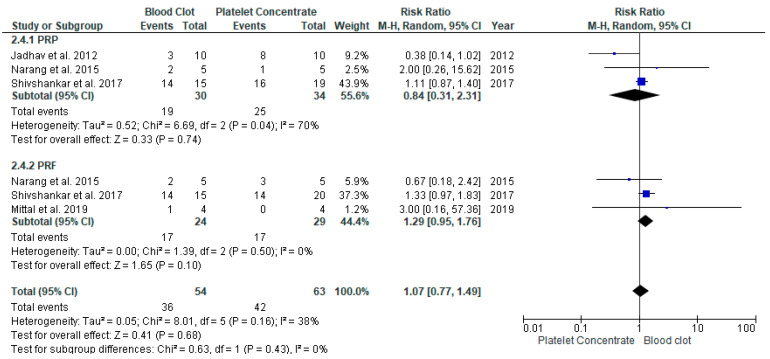
Meta-analysis of dentin wall thickness (DWT) in regenerative endodontic procedure (REP) using APC or BC.

Four studies [19,22,23,26] compared the DWT in REP between APC and BC. Data were pooled to assess the dentinal wall thickness (Figure 4). The overall risk ratio is 1.07, at 95% CI [0.77, 1.49] of achieving adequate dentinal wall thickness was found to be insignificant among these two group (*p* = 0.68). The heterogenicity between the study was moderate, at I^2^ = 38%.

#### 3.4.2. Increased Root Length in REP with APC Compared to BC in Young Immature Permanent Teeth

**Figure 5:**
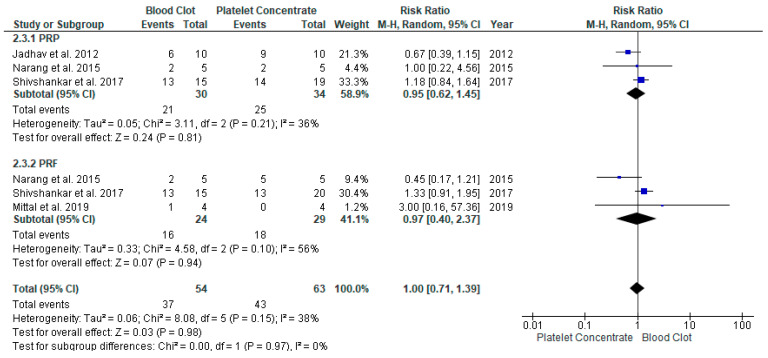
Meta-analysis of increased root length (IRL) in regenerative endodontic procedure (REP) using APC or BC.

Four studies [19,22,23,26] compared the effectiveness of APC to BC and assessed the increase in root length (Figure 5). The overall risk ratio was 1.00 with the 95% CI [0.71, 1.39] of achieving excellent/good root length found not to be significant among the two groups *p* = 0.95. The heterogenicity between the study was low, at I^2^ = 38%.

#### 3.4.3. Apical Closure Formation in REP with APC Compared to BC in Young Immature Permanent Teeth

**Figure 6:**
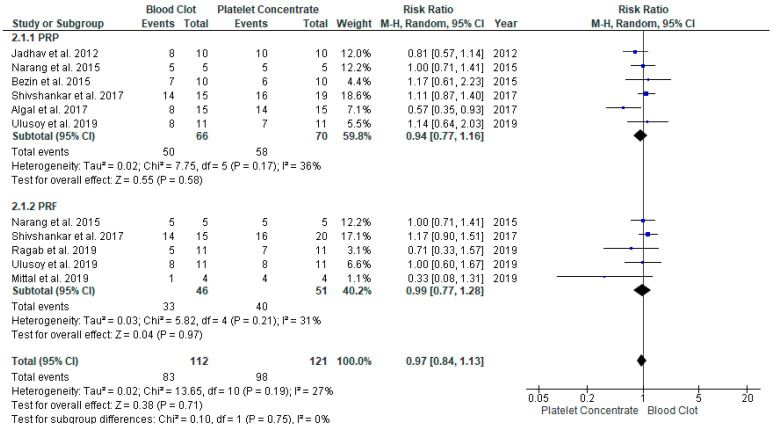
Meta-analysis of apical foramen width (AFW) in regenerative endodontic procedure (REP) using APC or BC.

Six studies [16,17,19,23,26,28] compared the apical closure of APCs to BC. Both the procedures showed no significant difference between the groups with a RR of 0.97 and 95% CI [0.84, 1.13], *p* = 0.19; this suggested a similar rate of apical closure at the end of follow-up (Figure 6). The heterogenicity between the study was low, at I^2^ = 30%.

#### 3.4.4. Vitality Response in REP with APC Compared to BC in Young Immature Permanent Teeth

**Figure 7:**
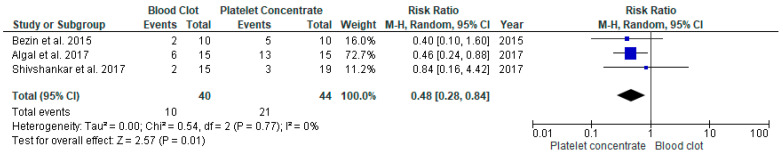
Meta-analysis of vitality response (VR) in regenerative endodontic procedure (REP) using APC or BC.

Three studies [16,17,23] compared the effectiveness of APC and BC. Both procedures had significant difference with RR 0.48, at 95% CI [0.28, 0.84], *p* = 0.01 (Figure 7). These findings suggests that positive vitality response at the end of follow-up was higher in the APC group. The heterogenicity between the studies was low at I^2^ = 16%.

#### 3.4.5. Success Rate of REP with APC Compared to BC in Young Immature Permanent Teeth

**Figure 8:**
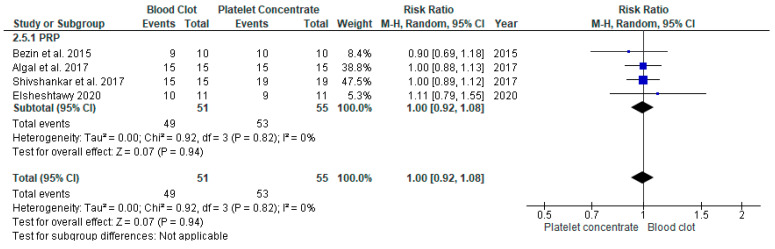
Meta-analysis of success rate in regenerative endodontic procedure (REP) using APC or BC.

Four studies [16,17,18,23] were pooled to assess the success rate. The overall risk ratio was 1.00 with a 95% CI [0.92, 1.08] and *p* = 0.96 (Figure 8). The success rate between both groups was found not to be statistically significantly different.

#### 3.4.6. Survival Assessment in Young Immature Permanent Teeth Undergone either REP or Apexification Procedure in Young Immature Permanent Teeth

**Figure 9:**
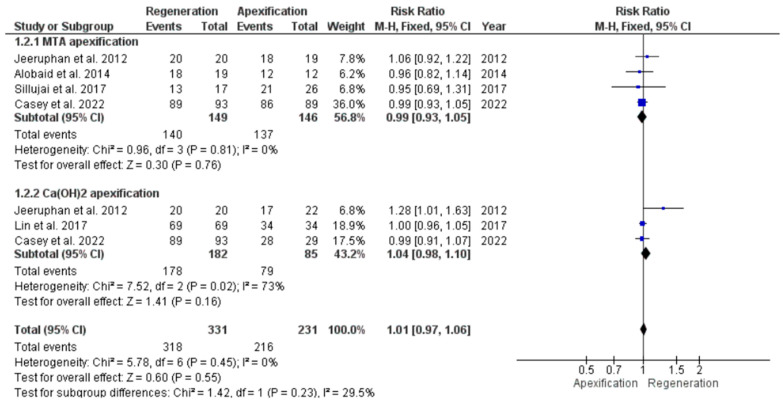
Meta-analysis of survival rate of young immature permanent teeth that underwent regenerative endodontic procedure (REP) or apexification procedure.

Five studies [39,41,42,45,46] were pooled to assess the survival rate. The procedures showed no significant difference with RR 1.01, at 95% CI [0.97, 1.06], *p* = 0.55 (Figure 9). These values suggest that both interventions led to a statistically similar rate of survival at the end of follow-up. The heterogeneity between the studies was low, at I^2^ = 0%.

However, a subgroup analysis observation was that apexification with MTA and REP exhibited a similar survival rate at RR 0.99, with 95% CI [0.93, 1.05], *p* = 0.76, I^2^ = 0%. In the same forest plot it was observed that the Ca(OH)_2_ apexification procedure had a low success rate compared to the MTA apexification procedure.

**Figure 10:**
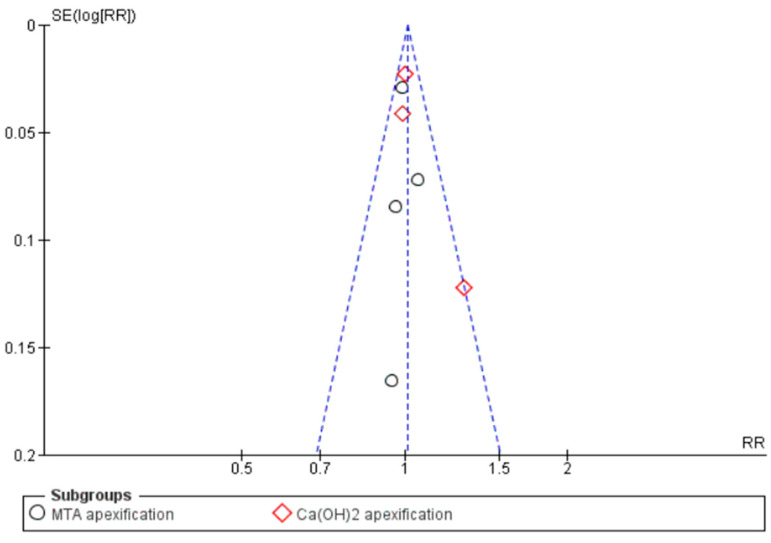
Funnel plot showing publication bias of studies on survival rate of young immature permanent teeth that underwent regenerative endodontic procedure (REP) or apexification procedure.

The funnel plot suggests low publication bias, with all studies placed within the inverted funnel (Figure 10).

#### 3.4.7. Comparison of Success Rate in Young Immature Permanent Teeth Treated with REP or Apexification Procedure

**Figure 11:**
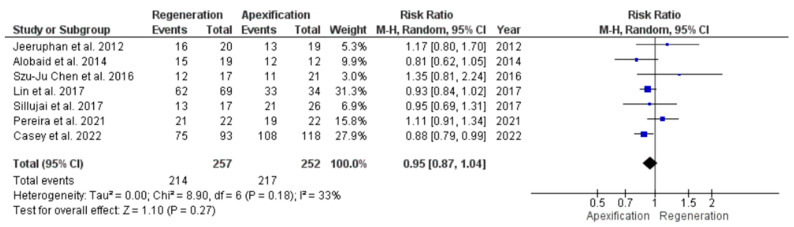
Meta-analysis of success rate in young immature permanent teeth undergoing regenerative endodontic procedure (REP) or apexification procedure.

Seven studies [39,41,42,44,45,46,47] were pooled to assess the success rate between two interventions. However both the procedures showed no significant difference with RR of 0.95, at 95% CI [0.87, 1.04], *p* = 0.27; suggesting similar success rates at the end of follow-up. The heterogeneity between the studies was low, at I^2^ = 33% (Figure 11).

**Figure 12:**
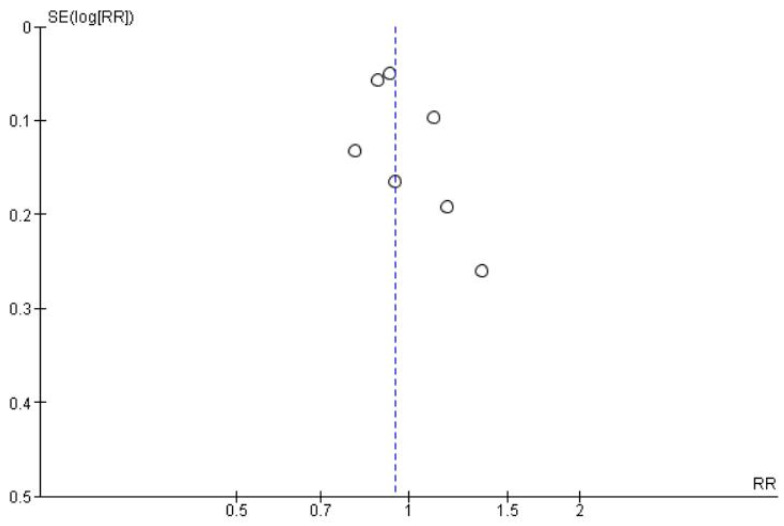
Funnel plot showing publication bias of studies on success rate in young immature permanent teeth undergoing regenerative endodontic procedure (REP) or apexification procedure.

The funnel plot suggests low publication bias, with all studies placed within the inverted funnel (Figure 12).

#### 3.4.8. Comparison of Increase in Root Length in Young Immature Permanent Teeth Treated with REP or Apexification Procedure

**Figure 13:**
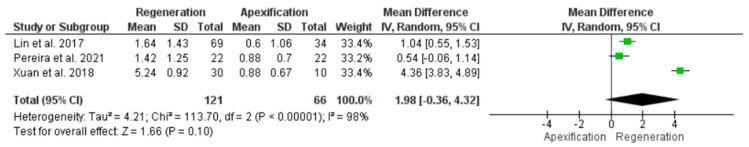
Meta-analysis of increase in root length (IRL) in young immature teeth treated with regenerative endodontic procedure (REP) or apexification procedure.

Three studies [39,40,44] were pooled to assess and compare the increase in root length. The increase in root length was significantly greater in the regenerative procedure compared to apexification, with a mean difference MD 1.98, 95% CI [-0.36, 4.32], *p* < 0.00001 (Figure 13). However, the heterogeneity between the studies was high, at I^2^ = 98%, questioning the reliability of the finding.

#### 3.4.9. Comparison of Decrease in Apical Foramen Width in Young Immature Permanent Teeth Treated with REP Or AEP

**Figure 14:**
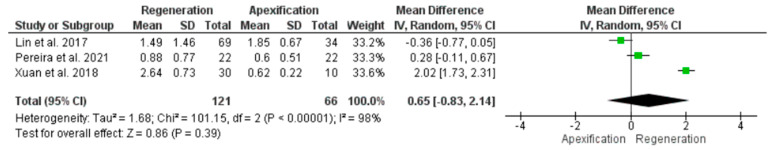
Meta-analysis of apical foramen width (AFW) in young immature teeth treated with regenerative endodontic procedure (REP) or apexification procedure.

Three studies [39,40,44] were pooled to assess and compare the decrease in apical foramen width. The decrease in apical foramen width was significantly greater in the REP compared to the apexification procedure with a mean difference (MD) of 0.65 at 95% CI [−0.83, 2.14], *p* < 0.00001 (Figure 14). However, the heterogeneity between the studies was high, at I^2^ = 98%, questioning the reliability of the finding.

## 4. Discussion

This systematic review was intended to analyze the various parameters that affect the survival of the immature necrotic tooth in the oral cavity after regeneration (REP) or apexification (AEP). The body of evidence for each comparison and outcome was assessed by considering the overall risk of bias in the included studies. The directness of the evidence, the inconsistency of the results, the precision of the estimates and the risk of publication bias were considered.

REP is based on tissue engineering, where a scaffold consisting of stem cells and essential growth factors support the proliferation and differentiation of stem cells [51]. An ideal natural scaffold should have a suitable porosity for cell seeding, potency to transport the nutrients, oxygen and waste, proper physical and mechanical strength, minimal inflammatory response and a similar biodegradable ability compared with the tissue regeneration process [52]. Blood clots (BC) and autologous platelet concentrates (APCs) are routinely used as scaffolds in REP [51]. BC is the process of forming a natural clot where the blood changes from a liquid to a gel. It has several advantages over alternative scaffolds, such as no allergic reaction, reduced cost and visiting time, convenience and comfort for patients. The clotting process involves many blood cells and clotting factors [51].

APCs are blood-derived products with an above-baseline concentration of platelets and an increased number of platelet-derived growth factors [53]. The principle of APC formation is the collection of the most active constituents of a small blood sample, which are plasma, platelets, fibrin, and leukocytes in most cases [54]. APCs are a cost-effective and useful in regenerative endodontics due to their high concentration of growth factors that induce migration, proliferation, and differentiation of stem cells, their dense fibrin matrix that serves as a stable scaffold and their bacteriostatic properties [55].

Platelet-rich plasma (PRP) is a gel with a high concentration of autologous platelets suspended in a small amount of plasma after centrifugation of the patient’s blood. The platelets in PRP play an essential role in treating the healing of damaged tissue due to the release of various growth factors such as PDGF, VEGF, IGF-1, FGF and EGF. The granules in platelets contain cytokines, chemokines and many other proteins that help stimulate proliferation and cellular maturation [56]. The platelet rich fibrin (PRF) is achieved with a simplified preparation, with no biochemical manipulation of blood. This technique does not require anticoagulants [57].

The teeth included for RET intervention were those that were affected by either trauma [58], secondary caries [59] or developmental anomalies [58]. Factors that can affect the outcome of RET are irrigation protocol, final rinsing of canal and intracanal medicaments (ICM). Six out of twelve studies [16,17,18,20,22] of the included clinical trials followed standardized irrigation protocol given by the American Association of Endodontists (AAE) and the European Society of Endodontics (ESE) [60]. The other six studies [16,17,19,21,23,24] did not follow the irrigation protocol religiously. The ideal concentration of NaOCl is 1.25%, but if a higher concentration is used, it reduces the viability of stem cells and their odontogenic/osteogenic differentiation [61]. EDTA reduces the deleterious effect of sodium hypochlorite and improves cell survival and differentiation [61]. It also liberates the growth factors present in dentin that positively affect stem cell adhesion, migration and differentiation [62]. Studies in which EDTA was not used as final irrigant also affected the outcome. The most preferred ICM used in RET is a triple antibiotic paste containing minocycline, ciprofloxacin and metronidazole, followed by calcium hydroxide paste [63]. AAE recommends 0.1 mg of TAP, but at high concentrations, it has a cytotoxic effect on stem cells and reduces mineralization [64] and when minocycline is included, it can cause significant tooth discoloration [65]. Overall, it can be concluded that RET is a successful intervention for the management of immature necrotic permanent teeth with high to moderate certainty.

The meta-analysis conducted in this systematic review concluded that APCs significantly improved apical closure and response to vitality pulp tests. In contrast, no significant difference between APC and BC was observed in root lengthening, dentin wall thickness or the success rate of immature, necrotic teeth treated with regenerative endodontics. This finding agrees with the outcome of other studies by Panda et al. [66]. The possible reasons could be due to intentional induction of bleeding from the periapical region and the formation of a blood clot into the root canal in the revascularization procedure of immature necrotic teeth acts as a scaffold supporting angiogenesis, providing a pathway for the migration of stem cells from the periapical area, and inducing pulp regeneration and maturation of the root [67]. Some vital pulp tissue and Hertwig’s Epithelial Root Sheath may remain in teeth with open apices and necrotic pulps. When the canal is sufficiently disinfected, the inflammatory process reverses, and these tissues may proliferate [68]. The second factor is the apex diameter. A tooth with an open apex allows the migration of mesenchymal stem cells into the root canal space, allowing the host cell homing to form new tissue in the root canal space. An apical opening of 1.1 mm in diameter or more is beneficial, with natural regenerative endodontic treatment occurring in approximately 18–34% of teeth with immature roots [68]. The third factor is the patient age. It is directly related to the stage of root formation and apical diameter; it is likely a modifying factor in regenerative endodontic procedures [69]. RET was capable of regenerating the pulp–dentine complex to restore the vitality of tissue damaged in the canal space and increase thee thickness of the canal walls to strengthen the fragile immature permanent teeth [70,71]. The possible reasons that APC performed better than BC in these two parameters could be difficulties in sensible evaluating because of the layered coronal seal over the BC scaffold [72].

Among APC, PRF had better outcomes in terms of AC and VPR. Possible reasons could be that PRF is collection of a dense and stable fibrin network [73] that allows a slower release of growth factors compared to PRP; PRP releases significantly more growth factors when compared to PRF during the first 15–60 min after clot formation. In a short time, high concentrations of bioactive molecules released by PRP could be responsible for the apparent beneficial effects over PRF. From these observations, it could be concluded that there is a trend of PRP showing better results than PRF in regenerative endodontic procedures. However, more clinical studies with large sample sizes are required to confirm or deny this trend over a long follow-up period [74]. The outcome of teeth in Apexification studies evaluated the outcome in terms of calcific barrier [30,32,34], periapical healing [30,34], and success rate [29,30,34]. The material used for apexification is Ca(OH)_2_ and MTA in both RCTs and NRCTs. The traditional method for the treatment of young, permanent, non-vital teeth is apexification. Traditionally, the approach has been to use calcium hydroxide (Ca(OH)_2_) to induce apexification after disinfection of the root canals in a conventional manner [75]. Ca(OH)_2_ is readily available, easy to use, relatively inexpensive and widely used in clinical procedures [10]. The disadvantages of traditional, long-term Ca(OH)_2_ therapy include variability in treatment time, the unpredictability of formation of an apical seal, difficulty in following up with patients and delayed treatment [76].

The traditional use of Ca(OH)_2_ to achieve apexification is being gradually replaced by mineral trioxide aggregate (MTA) as a one-step technique [51,52]. The advantages of using an apical plug include the requirement for fewer appointments to complete the treatment, more predictable apical barrier formation and reduced need for patient follow-up appointments [77].

The results showed that both materials had similar clinical success rates, radiographic success rates and apical barrier formation rates; there was no significant difference between these two groups. To obtain complete closure of the root apex, Ca(OH)_2_ based apexification procedure requires long-term application of the dressing material (from 3 to 24 months). However, MTA was associated with a significantly shorter time to achieve apical barrier formation than the calcium hydroxide [74]. The clinical protocol for apexification may involve one or multiple monthly appointments to place calcium hydroxide inside the root canal and eliminate the intracanal infection, which stimulates calcification and produces the apical closure [78]. A systematic review [79] evaluated the outcomes of the apexification method using Ca(OH)_2_ or MTA in young, immature permanent teeth. The authors found that the MTA barrier is a better procedure compared to Ca(OH)_2_ apexification, because it does not require many appointments and the conformation of the barrier does not require an external factor to develop, as it does with Ca(OH)_2_ apexification and pulp regeneration. These findings are in agreement with the present systematic review.

Calcium hydroxide can induce underlying tissues to produce large amounts of mineralized matrices. In the matrix attached to calcium, calcified foci induce calcification of the newly formed collagenous matrix. The high pH of calcium hydroxide also plays a vital role in inducing hard tissue formation [80].

The MTA can be placed as an apical plug with previous applications intracanal with Ca(OH)_2_ to produce the disinfection of the same [53,81], or even the MTA can be used as a material for canal filling. MTA is not bonded to dentin, but the interaction of calcium and hydroxyl ions components with a phosphate-containing synthetic body fluid results in the formation of apatite-like interfacial deposits [82].

This systematic review included studies that compared regeneration procedures and apexification procedures. Both the interventions are aimed at saving immature necrotic teeth. However regeneration is best attempted when the root formation is less than two-thirds [6] according Cvek’s classification.

The studies included compared both the interventions, involving the teeth with the apex open more than 1 mm. In this scenario, both regeneration and apexification have a similar outcomes. Overall, both interventions are comparable and successful.

The clinical outcome of teeth in RET versus APT studies was evaluated in terms of increase in root length [39,40], apical foramen width [40,41,45,46], periapical healing [39,45], survival rate [39,41,45,46] and successful rate [39,41,45,46,47]. The scaffold to initiate regeneration was BC, and apexification was calcium hydroxide or MTA.

Meta-analysis showed that the regeneration procedure resulted in significant improvement in root length and apical foramen width, but there was no significant difference concerning ‘overall outcomes’ (clinical and radiographic) and survival rate outcomes between revascularization and apexification.

Revascularization generates a new pulp-like tissue inside the root canal to restore the tooth physiology and significantly reduce the risk of tooth loss [10,12,70,71,83]. This could be the reason for revascularization to yield significantly better results in terms of root maturation than apexification, and to be slightly more effective in providing an increasing lateral dentinal wall thickness and promoting the continuation of dentin thickness and root width with a reduction of periapical radiolucency. However, further investigation is required into whether this increase in DWT is truly from dentin deposition or cementum-like and bone-like structures [84]. Another systematic review [85], evaluated the clinical, radiographic and functional retention outcomes in immature necrotic permanent teeth treated either with pulp revascularization or apexification after a minimum of three months to determine which one provides the best results. The authors found that although pulp revascularization procedures may increase root length and width, some attempts should be made to use standard methods to quantify the ‘real gain’ in root development because some X-ray distortions may overestimate its increase. Moreover, it was concluded that there is still a need to establish proper concentrations for root canal disinfectants that might enhance the survival of SCAP, but also reduce the microbial load and risk of reinfection. Based on their meta-analysis, the results do not favor one treatment modality over the other.

According to AAE [60], irrigation with 1.5% NaOCl followed by 17% EDTA and intracanal medicaments with either TAP in concentrations of 0.1–1 mg/mL or Ca(OH)_2_ with 1 mg/mL provide a higher survival of stems cells of the apical papilla (SCAP) that may play an essential role in root maturation. However, the treatment protocols adopted in the included studies comparing apexification with RET [53,56,58] did not use this proposed concentration. This could be the reason why reinfection occurred more in RET compared to apexification.

Only one study evaluated the reinfection post intervention and concluded that it was seen more in RET than in apexification. The possible reasons could be the use of higher concentrations of irrigating solutions that may be harming the SCAP, precluding a potential benefit of root maturation in both the interventions. Some failures were observed due to reinfection of the canal, perhaps due to residual bacteria in the root canal as effectively observed in histological analyses [86]. There is still a need for further investigation on this topic because most of the failures observed in these studies were due to persistent infection or reinfection.

In another systematic review [59] evaluating the clinical and radiographic outcomes for nonvital immature permanent teeth treated using RET, the authors found excellent success rates regarding tooth survival and periapical pathology resolution following RET. However, the results for more favorable outcomes, such as continued root growth, were uncertain. This study is also in agreement with our systematic review results.

Discoloration to the tooth was seen more in RET than in apexification [39]. Only one study [39] analyzed crown discoloration in the regeneration procedure. This study reported that 2 out of 19 teeth (10.5%) treated with BC revascularization presented crown staining. The possible reason could be the use of intracanal medication TAP containing minocycline.

Only one study [39] analyzed the root fracture in the apexification procedure. In this study, dens evaginatus (DE) premolar was analyzed, and Ca(OH)_2_ was used to create the calcific barrier at the apex. Of 21 patients, 2 had cervical fractures, and one had an apical fracture. The possible reason for this outcome could be the fact that DE frequently occurs on the lingual side of the buccal cusp, which is part of the functional cusp, and thus fractures easily when the occlusal force is exercised. In the same study [39], pulp canal obliteration was observed in RET. The possible reason could be internal replacement resorption during the hard tissue regeneration inside the root canal [87]. A longer follow-up period would be required to observe the results and whether this influences the dental treatment. However, this is the only study with a moderate risk of bias. Hence, the inference of this study should be analyzed with caution.

Out of 32 studies included in this review, 17 studies were randomized control trials; 3 had a low risk of bias, and 14 had a moderate risk of bias. Most of the studies failed to ensure concealment of allocation and blinding of the outcome assessment. In addition, due to the nature of the treatment, most studies found it impossible to ensure blinding of the patient and personnel because the patients receiving platelet concentrates knew which groups they were assigned since they were submitted to blood draw. In non-randomized control trials, there was uncertainty in defining the proper selection of participants in most studies, along with the classification of interventions and deviations from intended interventions in a few studies. Therefore, these reasons led to moderate to serious overall risk assessment.

## 5. Conclusions

Clinicians should consider employing the REP in cases when the root development is severely deficient, with insufficient dentine, and where the tooth’s prognosis is hopeless even with an apexification procedure. With moderate to high certainty, APCs used in the REP procedure significantly improved apical closure and response to vitality pulp tests. However, overall both APCs and BC showed similar successful outcomes in the regeneration procedure. In the apexification procedure with moderate certainty, it can be concluded that both MTA and Ca(OH)_2_ are equally effective in forming the calcific barrier. With moderate certainty, it can be concluded that both regeneration and apexification procedures are equally comparable interventions and result with similar overall outcomes.

## Figures and Tables

**Table 1:** Search strategy.

Search Strategy
#1 immature teeth/immature tooth/immature permanent tooth/immature permanent teeth/young permanent tooth/young permanent dentition
#2 pulp revascularization/pulp regeneration/pulp revitalization/PRF/PRP/blood clot
#3 apexification/calcific barrier/apical closure/root end closure/root apex closure/root end formation/root apex closure/apical plug/MTA plug
#4 survival rate/dentinal thickness/pulp vitality/root completion/successful rate/periapical healing/decrease in apical foramen width

**Table 2:** Inclusion and exclusion criteria for selecting studies in the systematic review.

Inclusion Criteria	Exclusion Criteria
Study design: Randomized controlled trials, clinical studies, observational studies (Retrospective study)	Case reports, comments, conference proceedings
Patients with immature necrotic permanent teeth	Studies experimenting on vital teeth
Studies in which either one of the interventions or both are compared	Animal studies, case reports, in vitro studies, laboratory studies
Articles published in English language	

**Table 3:** Data extraction from included studies – the clinical protocol.

Author	Etiology of Pulp Necrosis	Presence of Periapical Lesion	Instrumentation	Irrigation Method	Intracanal Medication	Recall Time (in Weeks)	Preparation Protocol of APC	Access Restoration
Alagl et al., 2017 [16]	Secondary to trauma/caries	Yes	No	2.5% NaOCl (20 mL), sterile saline (20 mL), and 0.12% CHX (10 mL), followed by 17% EDTA after 3 weeks	TAP	3	PRP was prepared according to the description by Dohan et al. [48]. PRP was combined with equal volumes of sterile solution containing 10% calcium chloride and sterile bovine thrombin (100 U/mL) to achieve coagulation.	NR
Bezgin et al., 2015 [17]	Secondary to trauma/caries	Yes	No	2.5% NaOCl (20 mL), sterile saline (20 mL), and 0.12% CHX (10 mL), followed by 5% EDTA (20 mL) after 3 weeks	TAP	3	PRP was prepared according to the description by Dohan et al. [48]. PRP was combined with equal volumes of sterile solution containing 10% calcium chloride and sterile bovine thrombin (100 U/mL) to achieve coagulation.	Final restoration was completed with white MTA (Angelus, Londrina, Brazil), reinforced GI cement (Ketac Molar Easymix; 3M ESPE, Seefeld, Germany) and composite resin (Filtek Supreme XT; 3M ESPE, St Paul, MN, USA)
Elsheshtawy et al., 2020 [18]	Secondary to trauma and Dens invaginatus	Yes	No	20 mL of 5.25% NaOCl. At recall, 20 mL of 2.5% NaOCl, followed by 20 mL sterile saline and 10 mL of 17% EDTA solution	TAP	NR	PRP was prepared according to Dohan et al. [48], after which concentrated platelet-rich plasma (cPRP) was prepared and introduced inside dry root canals using a sterile 30 G syringe. The canal was then backfilled with cPRP to a level just beneath the CEJ and left to clot for 10 min	MTA, using a layer of reinforced GI (Riva self-cure, SDI limited, Bayswater, Victoria, Australia), followed by resin composite (Filtek Z250 universal restorative, 3 mol L, 3M ESPE, St. Paul, MN, USA)
Jadhav et al., 2012 [19]	Secondary to trauma/caries	No	Minimal (#60H file)	2.5% NaOCl (copious irrigation)	TAP	NR	PRP: 8 mL of blood drawn by venipuncture of the antecubital vein was collected in a 10 mL sterile glass tube coated with an anticoagulant (acid citrate dextrose) and centrifuged at 2400 rpm for 10 min to separate PRP and platelet-poor plasma (PPP) from the red blood cell fraction. The topmost layer (PRP + PPP) was transferred to another tube and again centrifuged at 3600 rpm for 15 min to separate the PRP to precipitate at the bottom of the glass tube. This was mixed with 1 mL 10% calcium chloride to activate the platelets and to neutralize the acidity of acid citrate dextrose.	Resin-modified GI cement (Photac-Fill; 3M ESPE, St Paul, MN, USA)
Rizk et al., 2019 [20]	Secondary to trauma	Yes	No	20 mL 2% NaOCl for 5 min, followed by 20 mL 17% EDTA.	TAP	3	PRP was prepared according to the description by Dohan et al. [48]. PRP was combined with equal volumes of sterile solution containing 10% calcium chloride and sterile bovine thrombin (100 U/mL) to achieve coagulation. PRF: 10 mL blood was collected in a sterile tube without anticoagulant and centrifuged immediately for 10 min at a speed of 3000 rpm.	An MTA orifice plug extending 2–3 mm in the canal was used to seal the canal orifice then GI (GC America, Alsip, IL, USA) and composite (Z250, 3M ESPE) were applied to give an effective and durable seal
Ragab et al., 2019 [21]	Secondary to trauma	Yes	No	20 mL of 5.25% NaOCl followed by 20 mL sterile saline.	DAP	3	PRF was prepared by drawing 12 mL sample of whole blood intravenously from the patient’s right antecubital vein and centrifuged at 3000 rpm for 12 min.	MTA plus Light Cure GI cement
Mittal et al., 2019 [22]	Secondary to trauma/caries	Yes	Minimal (#30k file)	2.5% NaOCl (copious irrigation).	DAP	4	PRF was prepared by drawing 5 mL of venous blood from the patient, collected in a dried glass test tube, and centrifuged at 2700 rpm for 12 min.	GI cement followed by composite resin
Shivashankar et al., 2017 [23]	Secondary to trauma/caries	No	Minimal	5.25% NaOCl (copious irrigation).	TAP	3	NR	NR
Hazim Rizk et al., 2020 [24]	Trauma, Caries	Yes	No	20 mL of 2.5% NaOCl followed by 20 mL of 17% EDTA. At recall 20 mL sterile saline followed by 20 mL 17% EDTA solution	TAP	3	PRP and PRF was prepared according to Dohan and Choukroun (2007) [49] method.	MTA, using a layer of GI (GC America, Alsip, IL, USA) followed by composite (Z 250, 3 M ESPE)
Jiang et al., 2017 [25]	Trauma, Broken central cusp	Yes	NO	20 mL 1.25% NaOCl. At recall, 20 mL 17% EDTA.	Ca(OH)_2_ paste	2	NR	A layer of Filtek Z250 composite resin (3M ESPE, Irvine, CA; 3–4 mm) was placed over the capping material for the final restoration.
Narang et al., 2015 [26]	Secondary to trauma/caries	Yes	Minimal	2.5% NaOCl (copious irrigation)	TAP	4	NR	Resin-modified GI cement was placed extending 3–4 mm in the canal. Access cavity was sealed with composite (Clearfil Majesty, Kuraray Medical Inc., Tokyo, Japan).
Meschi et al., 2021 [27]	Trauma, Caries, Anatomic anomaly (dens invaginatus)	Yes	No	20 mL 1.5% NaOCl and subsequently with 20 mL saline. At recall, 30 mL EDTA 17% 1 mm short of the working length.	DAP	2	Blood samples were centrifuged. Fibrin clots were collected after centrifugation, and 2 of them were transformed into membranes after 5 min of pressure under a sterile glass plate.	Tooth was sealed by means of a GI lining and composite restoration.
Ulusoy et al., 2019 [28]	Secondary to trauma	Yes	No	20 mL 1.25% NaOCl. At recall, 2% CHX, saline and 1 mL 17% EDTA.	TAP	4	PRP: Citrated blood was centrifuged in a standard laboratory centrifuge PK 130 (ALC International; ColognoMonzese, Italy) for 15 min at 1250 rpm to obtain PRP without erythrocytes and leukocytes. PRF: 10 mL blood was collected in a sterile tube without anticoagulant and centrifuged immediately for 10 min at a speed of 3000 rpm (Andreas Hettich Group, Ltd., Tuttlingen, Germany).	MTA coronal barrier was sealed with a thin GI base, and final coronal restorations were placed at the same visit using acid etch composite resin.
Jayadevan et al., 2021 [33]	Trauma	No	Minimal (#80–120K file)	1.5% NaOCl solution (20 mL) followed by saline and 17% EDTA. Recall session, copious and gentle irrigation with saline and 20 mL of 17% EDTA.	TAP	4	A-PRF or PRF was freshly prepared using a centrifuge (R-8C Laboratory centrifuge, Remi Lab, Mumbai, India). For PRF, 10 mL of intravenous blood was drawn into a tube without anticoagulant and centrifuged at 2700 rpm for 12 min. For A-PRF, 10 mL of intravenous blood was drawn into a tube without anticoagulant and centrifuged at 1500 rpm for 14 min.	GI cement (GC, Fuji IX, GC India) was placed gently in a thickness of about 3–4 mm over the Biodentine and the access was temporized with Cavit. Post regenerative treatment consisted of non-vital bleaching or composite restoration. These procedures were performed after a period of one week.
Peng et al., 2017 [29]	Anatomic, Caries, Trauma	Yes	Minimal (#30K file)	5.25% NaOCl solution (20 mL)	TAP	1–4	NR	Conventional GI cement (Fuji IX, Fuji Corporation, Osaka, Japan) was placed over the blood clot at the level of CEJ, followed by phosphoric acid etching for 30 s, a single-bond adhesive agent, and placement of Filtek Z250 composite resin (3M ESPE, Irvine, CA, USA). Instead of GI cement, mixture of ProRoot MTA (Dentsply Tulsa Dental, Johnson City, TN, USA) with 3 mm thickness was placed at the level of the CEJ.
Lv et al., 2018 [30]	Dens evaginatus, Tooth fracture	Yes	Minimal (35 K-file)	20 mL of 1% NaOCl followed by 10 mL of 17% EDTA solution	TAP	4	PRF was prepared as described by Choukroun et al. [50]. Immediately before surgery, 5 mL of whole blood was drawn into 10 mL test tubes without anticoagulant reagent and was centrifuged at 400× *g* for 10 min. The PRF layer was separated using sterile scissors, and PRF clots were pressed into a membranous film with sterile dry gauze.	A 3-mm-thick layer of MTA was placed followed by a moist cotton pellet and Cavit. One week later, the Cavit was removed and replaced with a bonded resin restoration (Filtek Z350 XT: 3M ESPE Dental Products, St Paul, MN, USA).
Cheng et al., 2022 [31]	Secondary to trauma	No	Minimal or No	0.5–1.5% NaOCl and saline or NaOCl in combination with saline and 17% EDTA	TAP	2	CGF was prepared from the patient’s intravenous blood. After immediate differential centrifugation of blood, CGF was represented as the buffy coat in the middle layer. Then the CGF layer was separated using sterile scissors.	Teeth were restored with a bio-ceramic material [i.e., MTA (Dentsply Sirona, Ballaigues, Switzerland) or iRoot BP Plus (Innovative Bioceramix Inc, Vancouver, BC, Canada)] followed by various restorative materials.
Chueh et al., 2009 [32]	Trauma	Yes	No	2.5% NaOCl	Ca(OH)_2_ paste	1–2	NR	The access was sealed with temporary filling materials or resin.
Bonte et al., 2014 [34]	Trauma	Yes	No	Active 3% NaOCl	-	-	-	Composite resin
Santhakumar et al., 2018 [35]	Trauma and Dental caries	Yes	No	3% NaOCl followed by saline	TAP	3	A 5 mL blood sample was taken from the patient’s anticubital vein. The blood was centrifuged without anticoagulant at 3000 rpm for 10 min, and PRF gel was obtained at the bottom of the test tube and was removed with a sterile tweezer. After obtaining PRF gel, it was squeezed using especially designed PRF compression device to remove the excess fluid. The membrane obtained was cut linearly in the shape of root canal space for ease of placement.	Triple sealed with MTA (ProRoot MTA), type II GI cement (Fugi 2) and composite material (3M ESPE).
Kandemir Demirci et al., 2019 [36]	Trauma, Dens invaginatus, Caries	Yes	No	2.5% NaOCl solution. At recall, 2.5% NaOCl, 17% EDTA followed by 2% CHX	Ca(OH)_2_ powder mixed with saline	1	-	Bonded composite resin
Tek et al. 2021 [37]	Trauma	Yes	Yes	2.5% NaOCl solution. Recall 2.5% NaOCl solution followed by distilled water	Ca(OH)_2_ paste	1	-	Resin composite (3M ESPE Filtek Ultimate Seefeld, Germany)
Kinirons et al., 2001 [38]	Trauma	NR	No	NR	-	-	-	NR
Lin et al., 2017 [39]	Secondary to trauma/Dens evaginatus	Yes	Minimal (#25 K file)	20 mL 1.5% NaOCl, 0.9% physiological saline, 20 mL 17% EDTA	TAP	3	-	GI cement followed by composite resin
Xuan et al., 2018 [40]	Secondary to trauma	Yes	No	NR	NR	4	The pulp tissue for hDPSC isolation was harvested using standard sterile techniques. Autologous hDPSCs were obtained from the patient’s maxillary deciduous canine tooth.	NR
Alobaid et al., 2014 [41]	Secondary to Trauma	Yes	No	20 mL 17% EDTA	TAP	3	PRP and PRF were prepared according to the method of Dohan and Choukroun (2007) [49].	An MTA orifice plug extending 2–3 mm in the canal was used to seal the canal orifice then GI (GC America, Alsip, IL, USA) and composite (Z 250, 3 M ESPE) to give an effective and durable seal.
Casey et al., 2022 [42]	Secondary to trauma	Yes	Minimal	Varying concentrations of NaOCl, CHX, and/or EDTA	TAP	2	NR	Resin bonded restoration
Caleza-Jimenez et al., 2022 [43]	Trauma, Caries	Yes	No	1.5–2.5% NaOCl and 17% EDTA	TAP	2	NR	Composite restoration
Pereira et al., 2021 [44]	Trauma	No	Minimal	6% NaOCl, 2% CHX, saline solution, and EDTA 17% or Ca(OH)_2_ and 2% CHX gel	TAP	3	NR	Resin bonded restoration
Jeeruphan et al., 2012 [45]	Secondary to trauma/Caries	No	Minimal	5.25% NaOCl	TAP	3	NR	NR
Silujjai et al., 2017 [46]	Secondary to trauma/Caries/Dens evaginatus	Yes	No	1.5–2.5% NaOCl followed by 17% EDTA	Ca(OH)_2_ or TAP	NR	NR	MTA plus bonded restoration
Chen et al., 2016 [47]	Dens evaginatus	Yes	Minimal (#25 K file)	Copious 2.5% NaOCl	NR	NR	NR	NR

Legend: APC = autologous platelet concentrate; NR = not reported; NaOCl = sodium hypochlorite; CHX = chlorhexidine; EDTA = ethylene diamine tetra-acetic acid; DAP = double antibiotic paste; TAP = triple antibiotic paste; Ca(OH)_2_ = calcium hydroxide; GI = glass ionomer; PRP = platelet-rich plasma; cPRP = concentrated platelet-rich plasma; PPP = platelet-poor plasma; PRF = platelet-rich fibrin; CEJ = cementoenamel junction; MTA = mineral trioxide aggregate; hDPSC = human dental pulp stem cells.

**Table 4:** Data extraction from included studies for qualitative analysis – clinical evaluation parameters.

Author	Intervention	Type of Study	Comparative Group	Sample Size	Follow Up Time (in Months)	RA	Parameters to Assess Clinical Evaluation						
							DWT	IRL	AFW	AC	VR	PAH	BD
Alagl et al., 2017 [16]	REP	RCT	BC	15	12	CBCT	-	11.80 ± 3.28 mm	-	53.33%	53.33%	-	445.44 ± 153.54 HU
			PRP	15			-	12.14 ± 3.32 mm	-	93.33%	86.66%	-	485.88 ± 154.15 HU
Bezgin et al., 2015 [17]	REP	RCT	BC	10	18	IOPAR	-	12.6%	-	60%	20%	-	-
			PRP	10			-	9.86%	-	70%	50%	-	-
Elsheshtawy et al., 2020 [18]	REP	RCT	BC	11	12	CBCT	ICC = 1	ICC = 0.998	ICC = 1	-	-	-	-
			PRP	11			ICC = 0.997	ICC = 0.999	ICC = 0.998	-	-	-	-
Jadhav et al., 2012 [19]	REP	RCT	BC	10	12	IOPAR	S = 70% G = 30%	S = 40% G = 60%	-	S = 50% G = 30 E = 20%	-	S = 30%, G = 70%	-
			PRP	10			S = 20%, G = 50%, E = 30%	S = 10% G = 50% E = 40%	-	G = 30%, E = 70%	-	S = 10% G = 40% E = 50%	-
Rizk et al., 2019 [20]	REP	RCT	BC	13	12	IOPAR	-	0.68 ± 0.44 mm	2.2 ± 3.97 mm	-	-	-	58.96 ± 19.95 Grey
			PRP	13			-	1.48 ± 0.37 mm	2.49 ± 3.93 mm	-	-	-	65.08 ± 30.043 Grey
Ragab et al., 2019 [21]	REP	RCT	BC	11	12	IOPAR	-	14.8%	-	45.4%	-	80.5%	-
			PRF	11			-	12.8%	-	63.6%	-	70.2%	-
Mittal et al., 2019 [22]	REP	RCT	BC	4	12	IOPAR	100%	25%	-	25%	-	75%	-
			PRF	4			100%	0	-	100%	-	75%	-
Shivashankar et al., 2017 [23]	REP	RCT	BC	15	12	IOPAR	93.3%	86.7%	-	-	13.30%	2.07 ± 0.594 mm	-
			PRP	19			84.2%	73.7%	-	-	15.8%	1.32 ± 0.478 mm	-
			PRF	20			70%	65%	-	-	15%	1.85 ± 1.040 mm	-
Hazim Rizk et al., 2020 [24]	REP	RCT	PRP	13	12	IOPAR	-	1.48 ± 0.37 mm	0.97 ± 0.75 mm	-	-	-	65.08 ± 30.043 Grey
			PRF	12			-	1.24 ± 0.54 mm	1.003 ± 0.392 mm	-	-	-	53.44 ± 22.165 Grey
Jiang et al., 2017 [25]	REP	RCT	Without Bio-Gide	22	6	IOPAR	21.2 ± 19.5%	15.4 ± 13.6%	−55 ± 34%	-	18%	-	-
			With Bio-Gide	21			21.5 ± 22.5%	16.4 ± 13.6%	−65 ± 34%	-	33%	-	-
Narang et al., 2015 [26]	REP	RCT	MTA	5	18	IOPAR	0%	0%	-	0%	-	58%	-
			BC	5			50%	40%	-	66.67%	-	60%	-
			PRP	5			60%	99%	-	40%	-	98%	-
			PRF	5			20%	40%	-	60%	-	80%	-
Meschi et al., 2021 [27]	REP	RCT	REP-LPRF	13	36	CBCT	30%	0%	-	-	-	100%	-
			REP + LPRF	6			10%	10%	-	-	-	100%	-
Ulusoy et al., 2019 [28]	REP	RCT	BC	21	Until complete healing 10–49	IOPAR	14.91 ± 3.38 mm	7.15 ±1.39 mm	-	-	-	-	-
			PRP	18			19.01 ± 4.20 mm	4.74 ± 0.91 mm	-	-	-	-	-
			PRF	17			9.80 ± 3.03 mm	6.00 ± 1.57 mm	-	-	-	-	-
			PP	17			8.55 ± 3.55 mm	4.17 ± 1.33 mm	-	-	-	-	-
Jayadevan et al., 2021 [33]	REP	RCT	PRF	10	12	IOPAR	50%	80%				45.5%	
			APRF	11			91%	72%				40%	
Peng et al., 2017 [29]	REP	NRCT	Conventional GIC	32	12	IOPAR	26.3%	10.5%	-	-	-	-	-
			ProRoot MTA	28			30.7%	11.0%	-	-	-	-	-
Lv et al., 2018 [30]	REP	NRCT	BC	5	12	IOPAR	80%	80%	-	80%	100%	100%	-
			PRF	5			80%	80%	-	80%	100%	100%	-
Cheng et al., 2022 [31]	REP	NRCT	BC	32	16	IOPAR	F = 17.4 ± 16.4% L = 52.5 ± 24.8% Ci = 26.0 ± 37.3% A = 37.0%	F = 8.3 ± 11.7% L = 23.8 ± 18.1% Ci = 10.3 ± 16.6% A = 12.0%	F = 76.4 ± 30.9% L = 69.3 ± 43.9% Ci = 45.0 ± 37.7% A = 100.0%				
			CGF	30									
Chueh et al., 2009 [32]	REP	NRCT	MTA	8	6–108	IOPAR	-	87.5%	87.5%	-	-	-	-
			MTA + GP/GP/ Amalgam	15			-	93.33%	80%	-	-	-	-
Bonte et al., 2014 [34]	APP	RCT	MTA	15	12	IOPAR	-	-	76.5%	-	-	82.4%	-
			CH	15			-	-	50%	-	-	75.0%	-
Santhakumar et al., 2018 [35]	APP	RCT	PRF Gel	19	18	IOPAR	-	94.73%	-	-	100%	-	-
			PRF Membrane	19			-	89.47%	-	-	100%	-	-
Kandemir Demirci et al., 2019 [36]	APP	NRCT	MTA	39	12	IOPAR	-	-	74%	-	-	92%	-
			CH	34			-	-	79%	-	-	91%	-
Tek et al., 2021 [37]	APP	NRCT	Apical plug with MTA	10	12	IOPAR	-	-	-	-	-	50%	-
			Collagen sponge + apical plug with MTA	10			-	-	-	-	-	62.5%	-
Kinirons et al., 2001 [38]	APP	NRCT	CH in Newcastle	43	3	IOPAR	-	-	100%	-	-	-	-
			CH in Belfast	64			-	-	100%	-	-	-	-
Lin et al., 2017 [39]	REP vs. APP	RCT	BC	69	12	CBCT	82.60%	81.16%	-	65.21%	-	100%	-
			Vitapex paste	34			0%	26.47%	-	82.35%	-	100%	-
Xuan et al., 2018 [40]	REP vs. APP	RCT	hDPSC	20	12	CBCT	-	5.24 ± 0.92 mm	2.64 ± 0.73 mm	-	43.43 ± 0.86 mm	-	-
			CH	10			-	0.88 ± 0.67 mm	0.62 ± 0.22 mm	-	0.17 ± 0.16 mm	-	-
Alobaid et al., 2014 [41]	REP vs. APP	NRCT	BC	19	15–22	IOPAR	-	20%	10.2 ± −4.0%	-	-	-	-
			CH & MTA	12			-	12.5%	1.4 ± −3.2%	-	-	-	-
Casey et al., 2022 [42]	REP vs. APP	NRCT	BC	93	31–33	IOPAR	-	-	-	-	19%	-	-
			CH & MTA	118			-	-	-	-	0	-	-
Caleza-Jimenez et al., 2022 [43]	REP vs. APP	NRCT	BC	9	6–66	IOPAR		12.76%	34.57 ± 16.62%				
			MTA	9				0.29%	−3.36 ± 4.13%				
Pereira et al., 2021 [44]	REP vs. APP	NRCT	BC	22	12–30	IOPAR	0.21 ± 0.35 mm	1.42 ± 1.25 mm	0.88 ± 0.77 mm	-	-	95.45%	-
			MTA	22			0.03 ± 0.07 mm	0.88 ± 0.7 mm	0.6 ± 0.51 mm	-	-	86.36%	-
Jeeruphan et al., 2012 [45]	REP vs. APP	NRCT	BC	20	24	IOPAR	-	14.9%	28.2%	-	-	80%	-
			MTA	19			-	6.1%	0.00%	-	-	68%	-
			CH	22			-	0.4%	1.52%	-	-	77%	-
Silujjai et al., 2017 [46]	REP vs. APP	NRCT	BC	17	12–96	IOPAR	-	9.51 ± 18.14%	13.75 ± 19.91%	-	-	-	-
			MTA	26			-	8.55 ± 8.97%	−3.30 ± 14.14%	-	-	-	-
Chen et al., 2016 [47]	REP vs. APP	NRCT	CH, BC, MTA	17	12	IOPAR	-	94.12%	-	-	-	-	-
			CH, MTA	21			-	85.71%	-	-	-	-	-

Legend: REP = Regenerative Endodontic Procedure; APP = Apexification Procedure; RCT = Randomized clinical trial; NRCT = Non-randomised clinical trial; DWT = Dentin wall thickness; IRL = Increase in root length; AFW = Apical foramen width; AC = apical closure; VR = Vitality response; PAH = Periapical healing; BD = Bone density; BC = Blood clot; PRP = Platelet rich plasma; PRF = Platelet rich fibrin; PP = Platelet plug; MTA = Mineral trioxide aggregate; CH = Calcium hydroxide; hDPSC = Human dental pulp stem cells; RA = radiological assessment; IOPAR = Intraoral periapical radiographs; CBCT = cone-beam computed tomography; S = Satisfactory; G= Good; E= Excellent; ICC= Intraclass Correlation Coefficient; HU= Hounsfield units F= Fracture; L = Luxation; Ci= Combined injuries; A= Avulsion.

## Data Availability

Not applicable.

## Appendix A

The list of 21 articles [88,89,90,91,92,93,94,95,96,97,98,99,100,101,102,103,104,105,106,107,108] excluded from the review, with reasons for exclusion, is shown in Table A1.

**Table A1:** List of excluded studies after reading the full text.

Study	Reason for Exclusion
Alhaddad Alhamoui et al., 2014 [88]	In vitro study
Alkaisi et al., 2013 [89]	Animal study
El Arshy et al., 2016 [90]	Animal study
El-Tayeb et al., 2019 [91]	Animal study
Huang et al., 2013 [92]	Animal study
Peng et al., 2017 [93]	Chinese language
Jamshidi et al., 2018 [94]	In vitro study
Moradi et al., 2016 [95]	Animal study
Ok et al., 2015 [96]	In vitro study
Pagliarin et al., 2016 [97]	Animal study
Rafaei et al., 2020 [98]	In vitro study
Ritter AL et al., 2004 [99]	Animal study
Sogukpinar et al., 2020 [100]	In vitro study
Thibodeau et al., 2007 [101]	In vitro study
Valera et al., 2015 [102]	Animal study
Yang et al., 2018 [103]	Animal study
Yoo et al., 2014 [104]	Animal study
Zhang et al., 2014 [105]	Animal study
Zuong et al., 2010 [106]	Animal study
Beslot-Neveu et al., 2011 [107]	Study protocol
Bukhari et al., 2016 [108]	Case series
